# Rhizosphere microbial community enrichment processes in healthy and diseased plants: implications of soil properties on biomarkers

**DOI:** 10.3389/fmicb.2024.1333076

**Published:** 2024-02-29

**Authors:** Yong Deng, Wuyuan Kong, Xiaoming Zhang, Yi Zhu, Tian Xie, Ming Chen, Li Zhu, Jingzhao Sun, Zhihua Zhang, Chaoyong Chen, Chongwen Zhu, Huaqun Yin, Songqing Huang, Yabing Gu

**Affiliations:** ^1^Changde Tobacco Company of Hunan Province, Changde, China; ^2^School of Minerals Processing and Bioengineering, Central South University, Changsha, China

**Keywords:** soil-plant-microbe interactions, plant virus disease, rhizosphere microbes, biomarkers, co-occurrence network, soil properties

## Abstract

Plant health states may influence the distribution of rhizosphere microorganisms, which regulate plant growth and development. In this study, the response of rhizosphere bacteria and fungi of healthy and diseased plants compared to bulk microbes was analyzed using high-throughput sequencing. Plant adaptation strategies of plants under potato virus Y (PVY) infection have been studied from a microbial perspective. The diversity and community structure of bacteria and fungi varied between bulk and rhizosphere soils, but not between healthy and diseased rhizosphere soils. A LEfSe analysis revealed the significant differences between different treatments on bacterial and fungal community compositions and identified *Roseiflexaceae, Sphingomonas*, and *Sphingobium* as the bacterial biomarkers of bulk (BCK), healthy rhizosphere (BHS), and diseased rhizosphere (BIS) soils, respectively; *Rhodotorula* and *Ascomycota_unidentified_1_1* were identified as the fungal biomarkers of bulk (FCK) and healthy rhizosphere (FHS) soils. Bacterial networks were found to be more complex and compact than fungal networks and revealed the roles of biomarkers as network keystone taxa. PVY infection further increased the connectedness among microbial taxa to improve rhizosphere microbial community stability and resistance to environmental stress. Additionally, water content (WC) played an apparent influence on bacterial community structure and diversity, and pH showed significant effects on fungal community diversity. WC and pH greatly affected the biomarkers of bacterial rhizosphere communities, whereas the biomarkers of bulk bacterial communities were significantly affected by soil nutrients, especially for *Sphingobium*. Overall, the rhizosphere microbial community enrichment processes were different between healthy and diseased plants by changing the community compositions and identifying different biomarkers. These findings provide insight into the assemblage of rhizosphere microbial communities and soil physicochemical properties, which contributes to a deeper understanding of the establishment of an artificial core root microbiota to facilitate plant growth and bolstering resistance mechanisms. This knowledge contributes to a deeper understanding of the establishment of an artificial core root microbiota, thereby facilitating plant growth and bolstering resistance mechanisms.

## Introduction

Potato virus Y (PVY) is a significant constituent of the *Potyviridae* family, exhibiting the ability to infect over 170 species across the globe. The infection by PVY results in a wide range of symptoms, such as vein necrosis and systematic mottles, with necrotic ringspots reducing crop yield and quality substantially (Chen et al., 2017). Upon infection, plants activate their defense mechanisms by producing various resistant substances, including phytoalexin chitinase, peroxidase, and plant antitoxin to enhance their tolerance (Shah et al., 2017). Furthermore, the presence of PVY in a host organism leads to the observation of numerous morphological, physiological, and histological alterations (Hinrichs-Berger et al., 1999). However, the extent to which PVY infection affects the rhizosphere, a critical component of soil-plant interaction, is constrained. Prior studies have provided evidence of the impact of plant diseases or pests on the composition of rhizosphere microbiomes (French et al., 2021; Lazcano et al., 2021; Enagbonma et al., 2023). For instance, the introduction of the downy mildew pathogen to *Arabidopsis thaliana* leaves induces changes in the microbial communities within the rhizosphere (Berendsen et al., 2018). Similarly, the presence of the western corn rootworm, a pest that inflicts damage on maize plants, results in the proliferation of distinct microbial taxa in the rhizosphere, such as *Acinetobacter, Smaragdicoccus*, and *Aeromicrobium* (Benitez et al., 2017).

Plants establish direct contact with numerous microorganisms in the soil through the roots (Lu et al., 2006), leading to the observable differentiation between bulk and rhizosphere soils. The rhizosphere effect, a phenomenon in which plant roots attract and accumulate certain microorganisms from the bulk soil (Hein et al., 2008), plays a vital role in governing rhizosphere microbial communities. The rhizosphere, functioning as the primary site of soil-plant interaction, typically demonstrates a higher frequency of nutrient exchange and increased microbial activities in comparison to the bulk soil (Chaparro et al., 2014). The observed dissimilarity in microbial communities between the rhizosphere and bulk soil can be ascribed to root exudates, rhizosphere metabolism, and the root system's heightened selectivity. Consequently, the disparities in microbial communities are expected to result in variations in nutrient preferences and metabolic patterns of microbial communities (Lareen et al., 2016), thereby facilitating the suppression of phytopathogens and bolstering tolerance to environmental stress. Increasing evidence demonstrated the effect of numerous factors on the shifts in microbiomes of rhizosphere and bulk soil, including abiotic factors such as disease and insect pests (Yin et al., 2021), soil types (Lopes et al., 2021), plant species (Ahmad et al., 2022), and climate factors (Zhao et al., 2021). However, root-rot disease of *Zanthoxylum bungeanum* trees showed significant changes in the KEGG and CAZy functional profiles of microbiomes between the rhizosphere and bulk soils, rather than microbial diversity and community composition (Liao et al., 2022). These feedback explorations about the recruitment of rhizosphere microbes from bulk soil are vital to the ecological functions of terrestrial ecosystems.

Limited investigations have been observed regarding the impacts of PVY invasion on the plant microbiome, especially for microbial interactions and community assembly.

Recent research has demonstrated that environmental factors, encompassing both biotic and abiotic factors, conventionally govern the equilibrium between stochastic and deterministic assembly processes (Aguilar and Sommaruga, 2020; He et al., 2021). It has been ascertained that a harmonized stochastic and deterministic assembly process confers benefits in upholding a diverse ecosystem. Modifications in soil physicochemical properties, such as heightened soil salinity and pH levels, may exert a deterministic impact on the composition of soil bacterial communities (Yu et al., 2022). Conversely, optimal soil pH values and diminished salinity content may contribute to the stochastic nature of soil formation. Additionally, it has been observed that disease-induced modifications in plant performance can trigger a series of consequential changes in the rhizosphere environment, thereby significantly impacting the equilibrium between deterministic and stochastic factors within the rhizosphere microbiome (Liu et al., 2022). Despite the apparent recognition of this phenomenon, comprehensive testing and examination of its intricacies have been infrequently conducted. It is possible to propose the hypothesis that the invasion of pathogens in the rhizosphere microbiome has a deterministic impact on the compositional variability of said microbiome by altering plant performance. The occurrence of pathogen invasion is frequently accompanied by alterations in the diversity of rhizosphere microorganisms (Jiang et al., 2023). As a result, it can be deduced that the invasion of pathogens affects the microbial interactions that depend on the particular types and quantities of microorganisms that exist. Moreover, the evaluation of whether the bacterial microbiome of plant roots demonstrates dynamic universality, characterized by consistent interactions between microbes and their surroundings across hosts, or if each individual's microbiota adheres to its own distinct set of principles, is yet to be determined.

This study aimed to investigate the response of rhizosphere bacteria and fungi in healthy and diseased plants, as well as the bulk microbe, in relation to microbial diversity, structure, composition, co-occurrence network, and their correlation with soil physicochemical properties. Specifically, this research examined the disparities in (1) the diversity, composition, and structure of microbial communities in the rhizosphere and bulk soil between diseased and healthy conditions, (2) the network interactions among microbial genera and subnetworks based on biomarkers and their associated taxa, and (3) the relationship between microbial communities and soil physicochemical properties to understand the adaptation strategies of rhizosphere microbes under potato virus Y infection. This study places specific emphasis on the influence of PVY infection on the assemblies of rhizosphere microbiome communities and presents a unique viewpoint on the role of the microbiome in enhancing plant resistance against diseases.

## Materials and methods

### Collection of soil samples

The sampling was conducted in August 2022 at tobacco fields located in Changde city (between 29°13′30^′′^-29°59′19^′′^N and 110°28′40^′′^-110°58′30^′′^E, Hunan Province, China). Part of the plants in the sampling sites were infected by potato virus Y. Eight sample sites, naturally and randomly infected by PVY, were selected, and three soil samples were collected from each site, namely bulk soil, rhizosphere soil of a healthy plant, and rhizosphere soil of a diseased plant. A diseased plant that showed infected symptoms (the plant vein necrosis, leaf distortion, and stem necrosis) and a healthy plant were randomly selected at each sampling site. Rhizosphere soil samples were collected from both healthy (H) and diseased plants (I) by gently uprooting them and subsequently shaking off any excess soil from the roots by hand. The soil adhering to the root segment within the range of 0–4 mm from the root was identified as rhizosphere soil. The ridge soil, which was more than 20 cm away from the plant, was collected as bulk soil (C). In total, there were 24 soil samples collected in our study. A dry ice blanket was used to transport all samples to the laboratory. We divided soil samples into two parts, storing one at 80°C for microbial experiments and submitting the other to the School of Resources and Environment at Southwest University for measurement of soil properties. The pH value, water content (WC), organic matter (OM), total nitrogen (TN), alkali hydrolyzable nitrogen (AHN), total phosphorus (TP), available phosphorus (AP), total potassium (TK), and available potassium (AK) were tested following the methods outlined in previous studies (Gu et al., 2019, 2022).

### DNA extraction, amplicon sequencing, and data processing

Total DNA of rhizosphere and bulk soil samples (0.5 g of fresh soil from each sample) were extracted with the FastDNA SPIN Kit for soil (MP Biomedicals, Solon, OH, United States) following the manufacturer's instructions. The total DNA concentration and quality were measured using a NanoDrop 1000 spectrophotometer (Thermo Scientific, Waltham, United States). The primers 799F (5′-AACMGGATTAGATACCCKG-3′)/1115R (5′-AGGGTTGCGCTCGTTG-3′) were used to amplify the V5-V7 region of the bacterial 16S rRNA gene, and primer fITS7 (5′-GTGARTCATCGAATCTTTG-3′)/ITS4 (5′-TCCTCCGCTTATTGATATGC-3′) was used to amplify the fungal ITS2 region (Kembel et al., 2014; Deng et al., 2021). The polymerase chain reaction (PCR) production was used to perform paired-end sequencing using an Illumina Hiseq 2500 platform at MEGIGENE Biotechnology Co., Ltd. (Guangzhou, China). In the NCBI database, bacteria and fungi raw data were uploaded under PRJNA946037 and PRJNA946055.

The sequences were processed using QIIME2-2022.8 according to previously described methods (Zhang et al., 2022). In summary, after the elimination of adaptors and primer sequences, the raw sequences were assembled for each sample based on the distinctive barcode. The clean sequences, exhibiting a similarity of 97%, were then allocated to amplicon sequence variants (ASVs) utilizing DADA2 (Callahan et al., 2016), and their representative sequences were classified using the SILVA reference database (version 132) and the UNITE database (version 10.05) for bacteria and fungi, respectively (Kõljalg et al., 2005; Quast et al., 2012). After discarding singletons, the ASVs table was resampled for downstream analysis.

### Biomarkers identified by LEfSe analysis

The linear discriminate analysis effect size (LEfSe), which was implemented in the *microeco* package in R (version 4.0.0), was used to obtain the biomarkers for each treatment (Qu et al., 2020). Initially, a non-factorial parametric Kruskal-Wallis (KW) sum-rank test was employed to identify significant differences in abundance, with a significance threshold of 0.05. Subsequently, LEfSe was conducted using LDA to assess the impact of each component's (species) abundance on the observed differences. A logarithmic LDA score threshold of 3.0 was set to discriminate features.

### Network analysis

To examine the relationships between bacteria and fungi, a co-occurrence analysis was conducted using the SparCC method implemented in the *SpiecEasi* package (Friedman and Alm, 2012). The network was visualized using Gephi (version 0.9.2). Bacterial and fungal taxa at the genus level that were present at < 4 out of 8 sites were excluded from the network analysis. Correlations were calculated using SparCC, with a threshold of absolute correlation set at 0.3. The *P*-values were adjusted using the Benjamini–Hochberg procedure, and only those with *P*-values below 0.05 were retained. A range of network topological properties, including degree, modularity, betweenness centrality, and average path length, were computed in the R package *igraph*. The subnetworks based on biomarkers identified by LEfSe analysis and their associated nodes were selected by Cytoscape (version 3.8.1). The determination of keystone species within the networks involved the calculation of within-module connectivity (Zi) and among-module connectivity (Pi). Specifically, network hubs were identified as having Zi values ≥ to 2.5 and Pi values ≥ 0.62, module hubs had Zi values ≥ 2.5 and Pi values < 0.62, and connectors had Zi values < 2.5 and Pi values ≥ 0.62. In addition, the networks among ASVs were also constructed following the above steps, and similar changes in topological properties compared to networks at the genus level were found (Supplementary Table S1).

### Statistical analysis

The community alpha diversity indices, including the Shannon index, species richness, and Pielou's evenness index, were calculated using the *vegan* package. The differences in alpha diversity indices and soil properties among bulk, healthy rhizosphere, and diseased rhizosphere soils were tested using ANOVA. Principal coordinate analysis (PCoA) based on Bray-Curtis distance was performed and visualized the community structure differences. Analysis of similarities (ANOSIM) with the Bray-Curtis distance and 999 permutations was carried out by “*anosim*” in the *vegan* R package. Spearman's linkages between the microbial communities and soil physicochemical properties were analyzed based on a mantel test using the “*linkET*” package (Sun et al., 2022). All the plots were visualized using the “*ggplot*” package.

## Results

### Soil physical and chemical properties in bulk and rhizosphere soils

Soil physical and chemical properties showed significant differences between bulk and rhizosphere soils but not between healthy and diseased rhizosphere soils (Table 1). The contents of TN, AP, TP, and AK were found to be significantly elevated in the rhizosphere soil compared to the bulk soil, as determined using ANOVA (*p* < 0.05). However, no significant differences were observed between the healthy and diseased rhizosphere soils in terms of these parameters. Other properties, including WC, pH, OM, AHN, and TK, showed no significant differences among bulk, healthy rhizosphere, and diseased rhizosphere soils.

**Table 1:** ANOVA of bulk and rhizosphere soil physical and chemical properties among treatments based on Tukey's test.

**Soil properties**	**CK**	**HS**	**IS**
WC	13.475 ± 2.971a	17.063 ± 4.609a	16.825 ± 4.615a
pH	5.975 ± 0.406a	6.575 ± 0.780a	6.600 ± 0.566a
OM	17.631 ± 5.195a	20.938 ± 4.885a	25.362 ± 8.817a
TN	**1.150** **±0.367b**	**1.300** **±0.325ab**	**1.775** **±0.641a**
AHN	88.42 ± 31.07a	93.14 ± 26.86a	115.97 ± 37.00a
AP	**19.75** **±11.29b**	**97.13** **±69.44a**	**112.63** **±62.33a**
TP	**0.557** **±0.079b**	**1.096** **±0.416ab**	**1.406** **±0.777a**
AK	**392.6** **±191.2b**	**1009.4** **±515.6a**	**962.5** **±303.6a**
TK	15.887 ± 3.986a	14.925 ± 3.478a	17.338 ± 5.674a

Data are means ± standard errors. Different letters and bold words indicate statistically significant differences among treatments (p < 0.05). WC, water content (%); OM, organic matter (g/kg); TN, total nitrogen (g/kg); AHN, alkali hydrolyzable nitrogen (mg/kg); TP, total phosphorus (g/kg); AP, available phosphorus (mg/kg); TK, total potassium (g/kg); AK, available potassium (mg/kg); CK, bulk soil; HS, healthy rhizosphere soil; IS, diseased rhizosphere soil.

### Variations of bacterial and fungal community diversities

**Figure 1:**
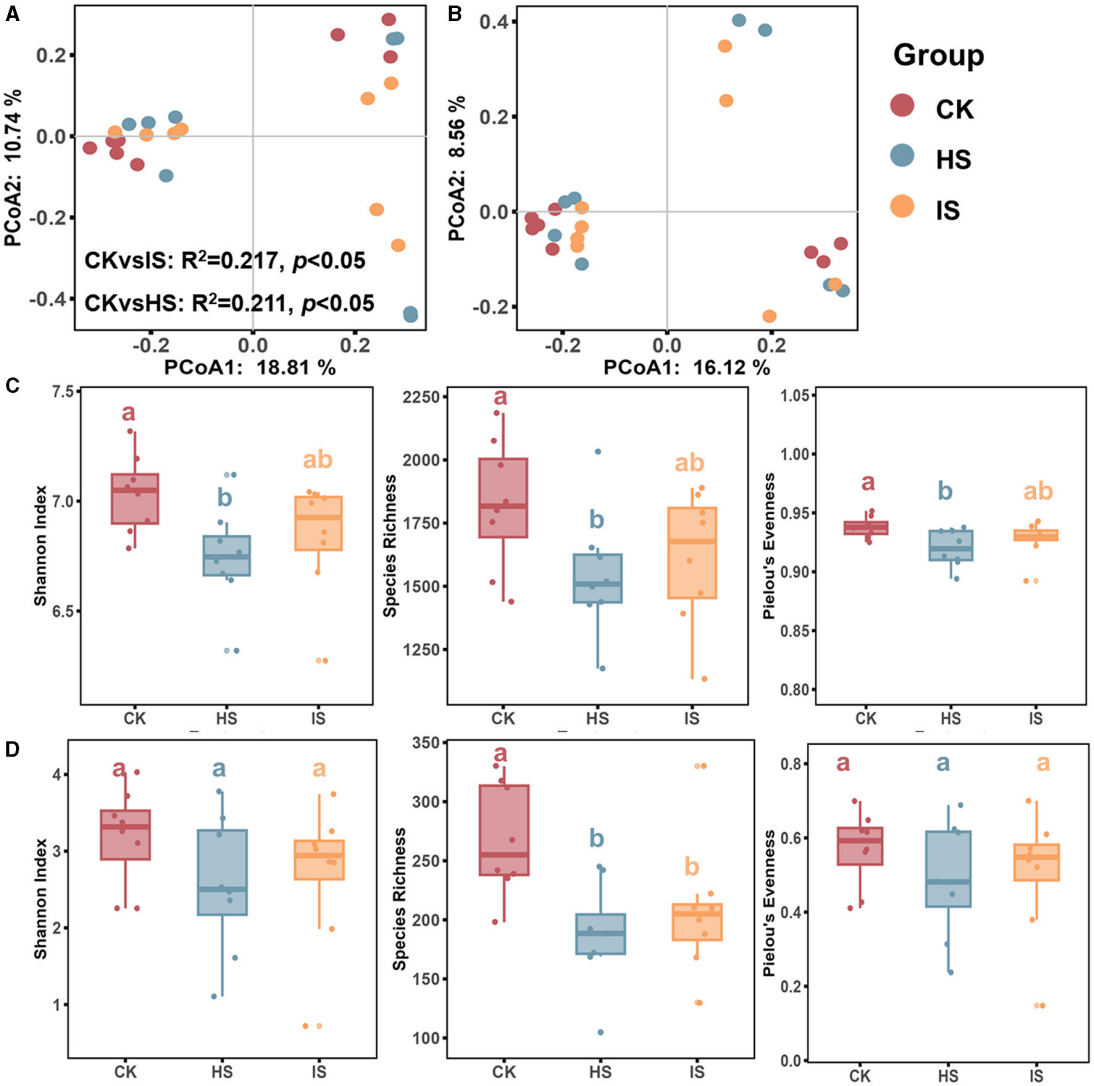
Microbial community diversity analysis in rhizosphere and bulk soils. PCoA of bacterial **(A)** and fungal **(B)** communities of bulk soil (CK), healthy rhizosphere soil (HS), and diseased rhizosphere soil (IS). ANOSIM analysis based on Bray-Curtis distance was conducted to reveal the community dissimilarity among CK, HS, and IS. Differences between rhizosphere and bulk soil bacterial **(C)** and fungal **(D)** community diversity indices, including the Shannon Index, species richness, and Pielou's evenness index. Different letters indicated a significant difference based on one-way ANOVA (Fisher's test) with *P* < 0.05.

Both PCoA and ANOSIM analyses revealed significant differences in the beta-diversities of bacterial communities between the bulk and rhizosphere soils (ANOSIM, *P* < 0.05) (Figure 1A). Specifically, the beta-diversity of bulk soil bacterial community (BCK) differed significantly from that in healthy rhizosphere soil (BHS, R^2^ = 0.211, *P* = 0.04) and diseased rhizosphere soil (BIS, R^2^ = 0.217, *P* = 0.03), whereas no significant difference was observed between BHS and BIS. ANOSIM analysis results showed that the fungal community beta-diversities among bulk and rhizosphere soil could not be significantly distinguished (*P* > 0.05) (Figure 1B). The alpha-diversity of the microbial communities, as measured by the Shannon index, species richness, and Pielou's evenness index, was calculated. An ANOVA analysis revealed a significant difference among the CK, HS, and IS groups (Figures 1C, D). Specifically, the bacterial community alpha diversity in the BCK was significantly higher than in the BHS. However, there was no significant difference in alpha diversity between the BIS and BCK/BHS (Fisher's test, *P* < 0.05). In terms of the fungal communities, the species richness in FCK was significantly higher than in the FHS and FIS groups (Fisher's test, *P* < 0.05).

### Biomarkers differentiating bulk and rhizosphere soil samples

**Figure 2:**
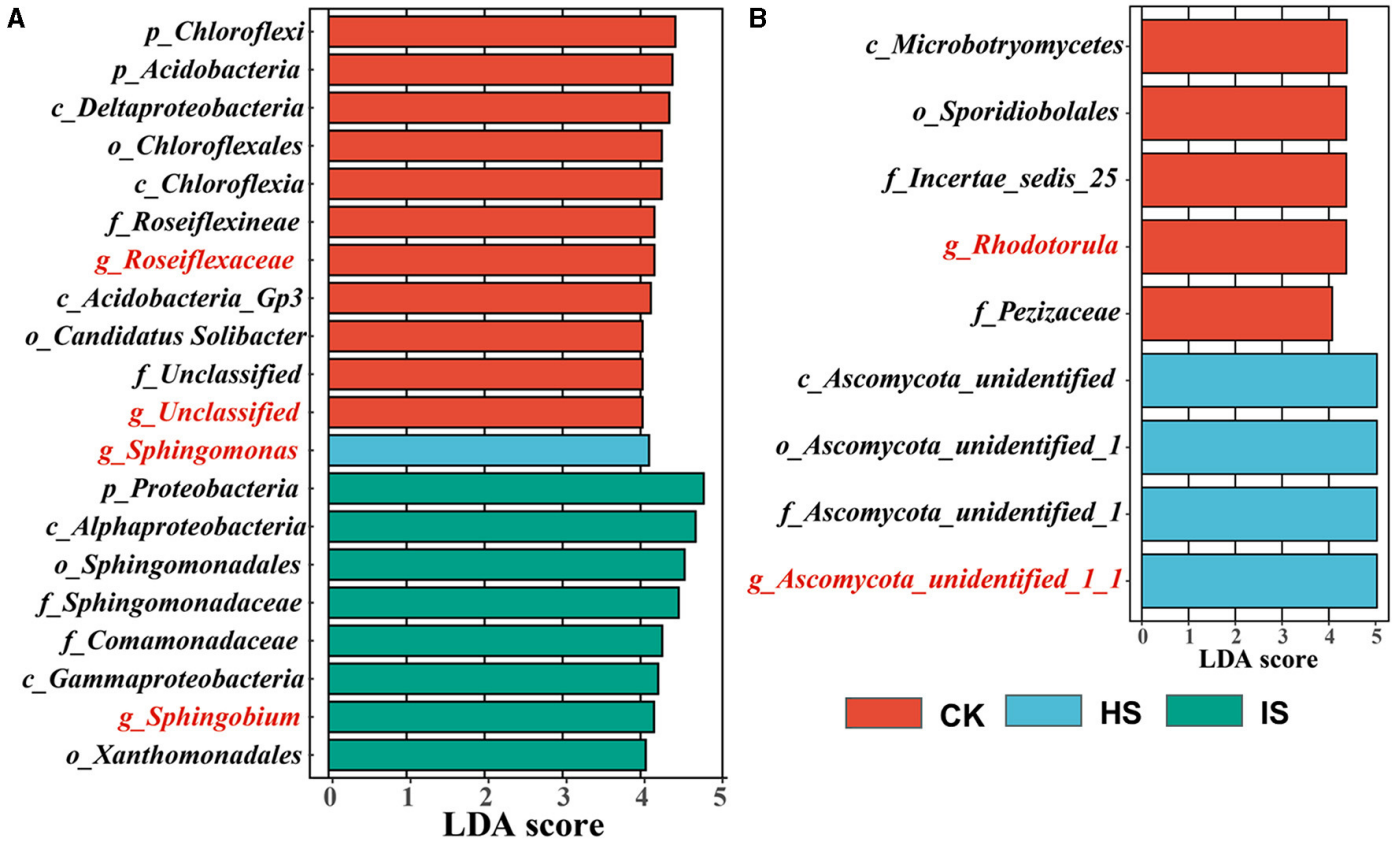
Taxa identified by linear discriminant effect size (LEfSe) analysis that explains the differences of bacterial **(A)** and fungal **(B)** communities among bulk soil (CK), healthy rhizosphere soil (HS), and diseased rhizosphere soil (IS). The threshold on the logarithmic LDA score for discriminative features was set to 4 (*P* < 0.05).

The LEfSe analyses were carried out to identify which taxa define the bacterial and fungal communities in CK, HS, and IS (Figure 2). There were 223 and 43 taxa found to be unique in the bacterial and fungal communities (*P* < 0.05), respectively. Accordingly, 11 out of 20 bacterial taxa were identified as the top bulk soil taxa with LDA of >4.0, whereas IS and HS soils had 8 and 1 taxa, respectively. The top bacterial taxa at the genus level in CK were *Roseiflexaceae*, which belonged to the phyla *Chloroflex*, and unclassified taxa belonging to the phyla *Acidobacteria* (LDA > 4.0). The top taxon in HS was *Sphingomonas*, which belonged to *Proteobacteria*. The top taxon in IS was *Sphingobium*, which belonged to *Proteobacteria*. For fungal communities, five out of nine fungal taxa were identified as the top bulk soil taxa, whereas four taxa were found in HS soil. The top fungal taxa associated with CK at the genus level were *Rhodotorula*, belonging to the phyla *Basidiomycota*, and *Pezizaceae* family, belonging to the phyla *Ascomycota*. The top taxon associated with HS was *Ascomycota* at the phylum level. For the IS soil communities, the defining taxa were *Guehomyces* belonging to the phyla *Basidiomycota* (LDA = 2.245). Therefore, *Roseiflexaceae, Sphingomonas*, and *Sphingobium* were identified as the bacterial biomarkers of CK, HS, and IS, respectively; *Rhodotorula* and *Ascomycota_unidentified_1_1* were identified as the fungal biomarkers of CK and HS.

### Network analysis of bacterial and fungal communities at the genus level

**Figure 3:**
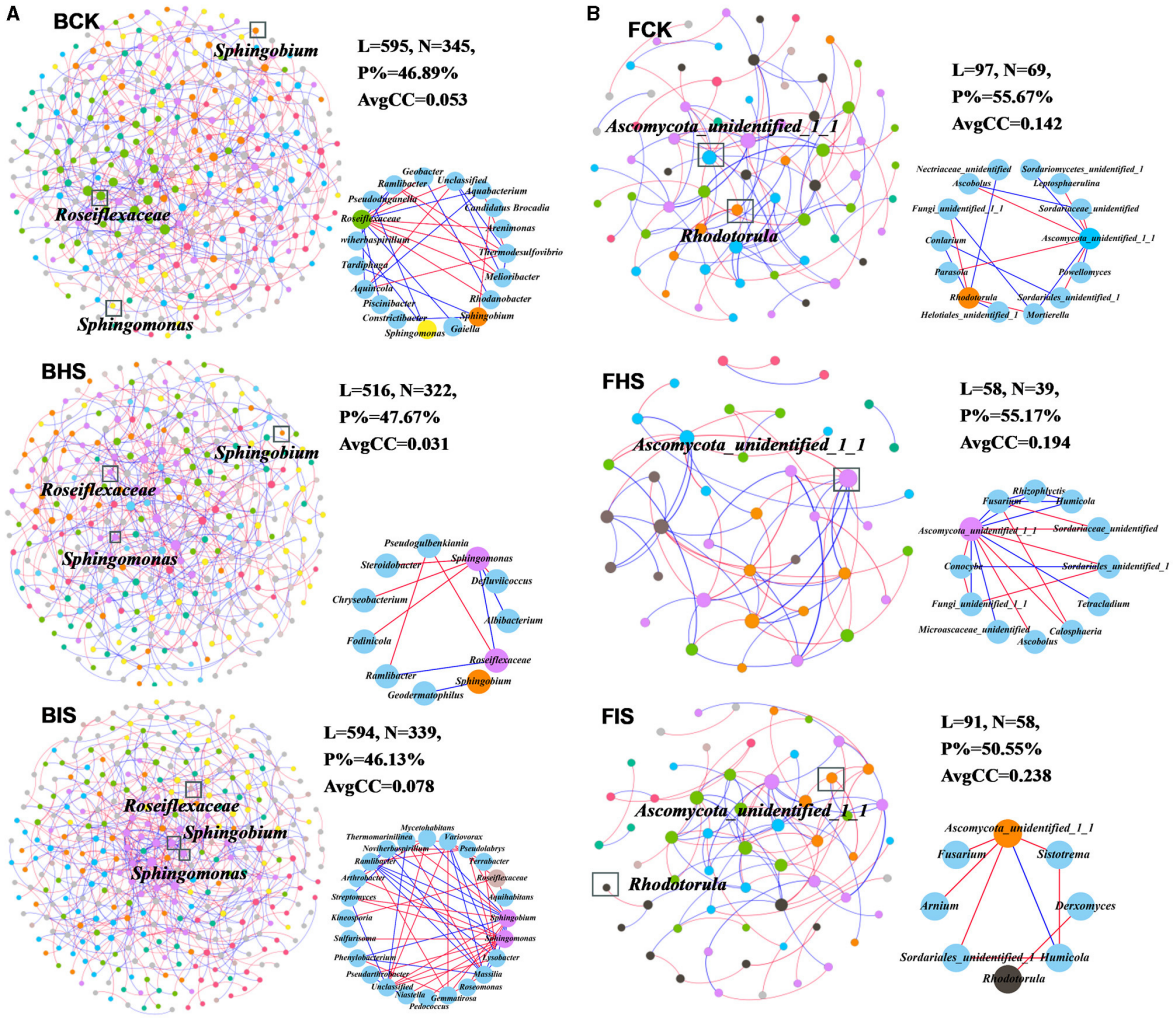
Overall networks and sub-networks of microbial communities at the genus taxonomic classification level. **(A)** Bacterial networks of bulk soil (BCK), healthy rhizosphere soil (BHS), and diseased rhizosphere soil (BIS). **(B)** Fungal networks of FCK, FHS, and FIS. Sub-networks were conducted based on biomarkers (*Roseiflexaceae, Sphingobium*, and *Sphingomonas* in bacterial communities and *Ascomycota_unidentified_1_1* and *Rhodotorula* in fungal communities) and their associated nodes. Nodes were colored by module assignment. The red line represented negative links between nodes, and the blue line represented positive links.

To explore the co-associated interactions of microbes in bulk soil, healthy rhizosphere soil, and diseased rhizosphere soil, a Spiec-Easi network analysis at the genus level was conducted (Figure 3), and the network topological properties of these networks in each soil compartment were calculated. For bacterial networks, the node numbers, link numbers, degree, and clustering coefficient of BHS were lower than those of BCK and BIS (Table 2), and the BHS network had a higher positive link proportion. In addition, these topological properties among BCK and BIS were similar. We also analyzed the sub-networks of biomarkers (*Roseiflexaceae, Sphingobium*, and *Sphingomonas*) and their associated nodes. *Roseiflexaceae* showed 11 links with other nodes, including six negative links and five positive links in the BCK network; *Sphingobium* had two positive links; and *Sphingomonas* had three positive links within four links. In the BHS network, *Roseiflexaceae* had two positive links within three links with other nodes, *Sphingobium* had one positive link, and *Sphingomonas* had five negative links within six positive links. In the BIS network, *Roseiflexaceae* had three negative links with other nodes, *Sphingobium* had six negative links within 13 links, and *Sphingomonas* had eight negative links within nine links. Furthermore, *Roseiflexaceae* and *Sphingomonas* worked as connectors in the overall networks of the BIS and BHS networks, whereas *Sphingomonas* worked as connectors in the BCK networks. Fungal networks were smaller, with fewer nodes and links compared to bacterial networks. Similar to bacterial networks, nodes and links in the FHS network were the smallest. The topological properties of the FIS network, including degree and clustering coefficient, were the highest compared to those of the FCK and FHS networks, suggesting that FIS was the most compact. For subnetworks based on biomarkers and their associated nodes (*Ascomycota_unidentified_1_1* and *Rhodotorula*), the nodes and links were the smallest in FIS and the largest in FHS. Interestingly, more than half of the links associated with keystone taxa (*Ascomycota_unidentified_1_1* in FHS and *Rhodotorula* in FCK) in the FCK and FHS subnetworks were positive, whereas almost all the links associated with *Ascomycota_unidentified_1_1* and *Rhodotorula* in the FIS network were negative. Furthermore, *Ascomycota_unidentified_1_1* and *Rhodotorula* both played the role of connectors in the FCK network, and *Ascomycota_unidentified_1_1* also worked as connectors in the FHS and FIS networks.

**Table 2:** Topological properties of bacterial and fungal networks in bulk, healthy rhizosphere, and diseased rhizosphere soils.

**Treatments**	**BCK**	**BHS**	**BIS**	**FCK**	**FHS**	**FIS**
Nodes number	345	322	339	69	39	58
Links number	595	516	594	97	58	91
Average degree	3.449	3.205	3.504	2.812	2.974	3.138
Average path length	4.936	5.113	4.785	3.941	3.018	3.390
Average clustering coefficient	0.053	0.031	0.078	0.142	0.194	0.238
Betweenness centralization	0.107	0.136	0.122	0.190	0.244	0.162
Degree centralization	0.031	0.040	0.052	0.091	0.211	0.120
Modularity	0.590	0.594	0.602	0.598	0.461	0.468

BCK, bulk bacterial network; BHS, healthy rhizosphere bacterial network; BIS, diseased rhizosphere bacterial network; FCK, bulk fungal network; FHS, healthy rhizosphere fungal network; HIS, diseased rhizosphere fungal network.

### Correlation between bulk and rhizosphere soil properties and microbial communities

**Figure 4:**
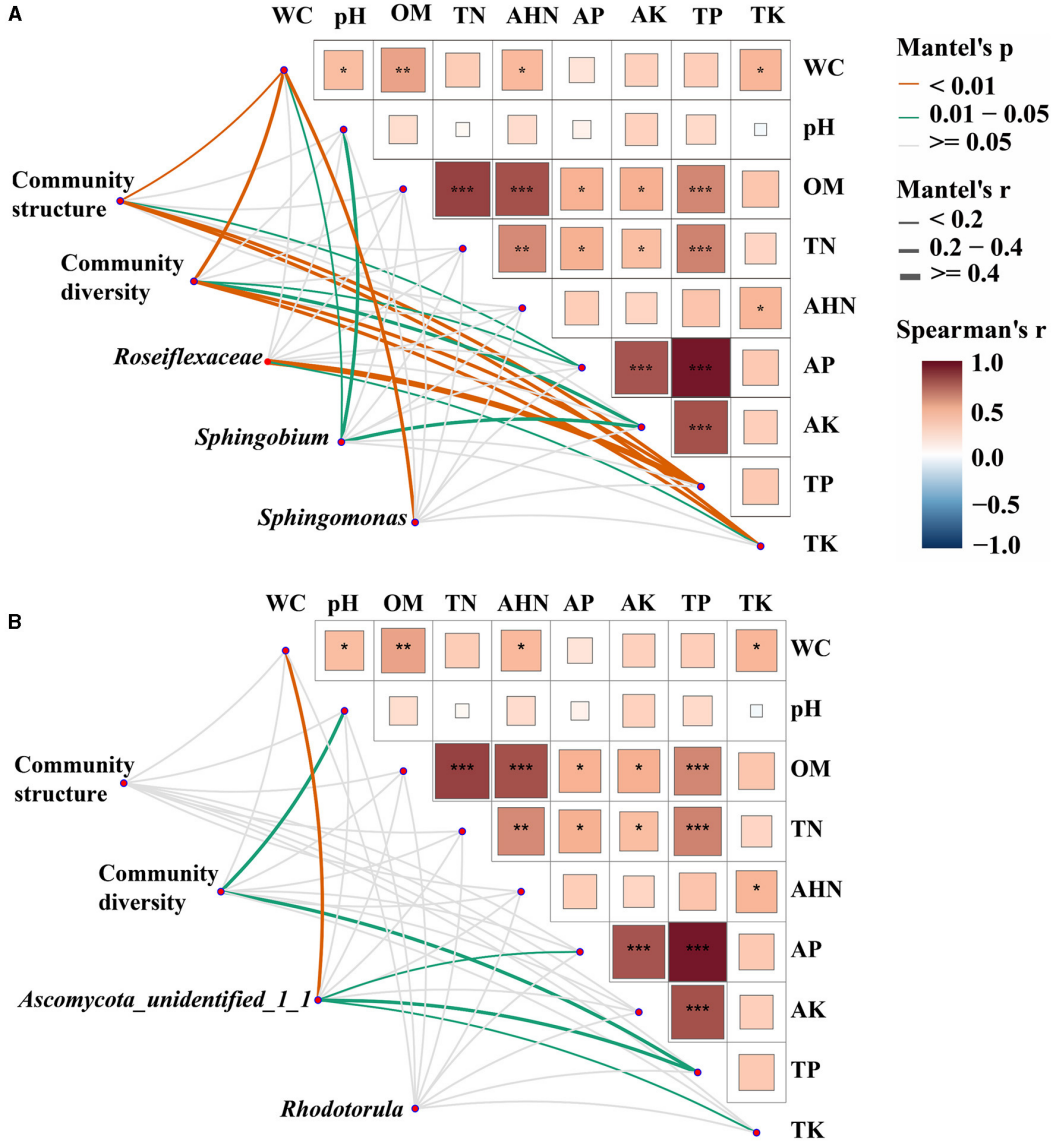
Environmental drivers of bacterial **(A)** and fungal **(B)** communities. Pairwise comparisons of soil physicochemical properties were shown, with a color gradient denoting Spearman's correlation coefficients. Microbial community structure (PCoA based on Bray-Curtis distance), community diversity indices (Shannon index, species richness, and Pielou's evenness index), and the relative abundance of biomarkers (*Roseiflexaceae, Sphingobium*, and *Sphingomonas* in bacterial communities and *Ascomycota_unidentified_1_1* and *Rhodotorula* in fungal communities) were related to each soil physicochemical property through partial Mantel tests. Edge width corresponds to Mantel's r statistic for the corresponding distance correlations, and edge color denotes the statistical significance based on 9,999 permutations. *p < 0. 05; **p < 0.01; ***p < 0.001.

Mantel tests were employed to ascertain the biomarkers that exert an influence on the bacterial and fungal communities in both bulk and rhizosphere soils (Figure 4). It was observed that the bacterial communities exhibited a greater susceptibility to soil properties when compared to the fungal communities. Specifically, WC, AP, TP, and TK were identified as significant factors impacting the structure and diversity of bacterial communities (*p* < 0.05). In fungal communities, pH and TP had significant effects on community diversity (*p* < 0.01), and there was no factor found to significantly affect community structure. Furthermore, the biomarkers of both bacterial and fungal communities were discovered to be influenced by the fluctuations in soil properties. The variations of *Roseiflexaceae* among bulk soil, healthy rhizosphere soil, and diseased rhizosphere soil were significantly influenced by TP and TK (*p* < 0.05), WC, pH, and AK were the key factors affecting the relative abundance of *Sphingobium* (*p* < 0.01), and WC was the key factor affecting *Sphingomonas* (*p* < 0.05). The fungal biomarkers *Ascomycota_unidentified_1_1* were significantly affected by WC, AP, TP, and TK, whereas no significant key factor was found for *Rhodotorula* genera (*p* < 0.05). Overall, bacterial and fungal communities had different key drivers, and bacterial communities had more driving factors than fungal communities.

## Discussion

### Differences in the diversity, composition, and community between bacteria and fungi in bulk soil, healthy rhizosphere soil, and diseased rhizosphere soil

The diversity of microbial communities in the rhizosphere is crucial to soil nutrient cycling and many plant processes (Lebeis, 2015). In agreement with most previous studies, rhizosphere soil microbial community diversities were significantly decreased compared with bulk soil (Bakker et al., 2015). Significant differences in community structure and a decline in community diversity were observed in this study. However, the microbial community diversity and structure of rhizosphere soils showed no significant correlation with the plant's healthy state in this study. The presence of plant diseases, including root-rot disease (Liao et al., 2022), *Verticillium* wilt (Wei et al., 2019), and ginseng rusty root (Bian et al., 2020), has been observed to induce alterations in the microbiomes of the root endosphere, rhizosphere, and soil. Different types of pathogens might be possible reasons for the different results of this study compared to previous studies. Previous studies usually focused on bacterial and fungal pathogens, whereas viruses are the pathogen for PVY disease. The transmission modes and infection types were different among bacterial, fungal, and virus-pathogen diseases. The infection of viruses into plants typically does not result in immediate mortality but rather induces modifications in their growth and developmental mechanisms, leading to alterations in plant pigmentation or morphology, commonly referred to as discoloration and deformity (Koziel et al., 2021). Thus, the effects of PVY infection on the rhizosphere might be relatively delayed compared with bacterial and fungal diseases. Differences among bacterial and fungal communities under PVY infection were also observed and can be attributed to different responses to soil physicochemical properties. However, there were no significant changes in community structures between bulk soil and rhizosphere soil. Several previous studies have shown similar results (Ai et al., 2018; Xue et al., 2022).

In this study, we found the different community compositions of bulk, healthy rhizosphere, and diseased rhizosphere soils. *Roseiflexaceae, Sphingomonas*, and *Sphingobium* were found to be the biomarkers of the BCK, BHS, and BIS communities, respectively. The genus *Roseiflexaceae* has been identified as a biomarker for the soil microbial community's yield, functioning as chemoautotrophic bacteria involved in carbon dioxide assimilation and significantly influencing the growth of *Eichhornia crassipes* (Wang et al., 2022). The genera *Sphingomonas* and *Sphingobium* are commonly found in rhizosphere soil and have been recognized as beneficial microbes that positively impact soil nutrient cycling. *Sphingobium* has been associated with carbon and nitrogen cycling in soil (Videira et al., 2009), while *Sphingomonas* has been reported to adapt to neutral pH and possess nitrogen-fixing capabilities. An increased abundance of *Sphingomonas* and *Sphingobium* in the rhizosphere could result in improved conditions for crop growth. More importantly, *Sphingomonas* has the biocontrol potential of plant disease (Innerebner et al., 2011). Thus, enrichment of *Sphingomonas* could be regarded as a potential defensive mechanism against plant diseases. An unclassified genus belonging to the *Ascomycota* phyla and the genus *Rhodotorula* were also identified as the biomarkers in the fungal communities of FHS and FCK, respectively. *Rhodotorula* was a fungal genus with a higher relative abundance in field soil (Tan et al., 2021). *Ascomycota* phyla was reported to be the dominant root-associated fungus (Peng et al., 2023), which was highly adaptable and actively involved in nutrient cycling (Egidi et al., 2019). Furthermore, *Ascomycota* possesses essential genes responsible for encoding cellulolytic enzymes and facilitating the carbon conversion process (Hannula et al., 2012). Therefore, bacterial and fungal community compositions manifest the adaptive traits of plants in diverse environments and contribute to the facilitation of crucial ecological processes through the regulation of the abundance of these prevailing microorganisms.

### PVY infection affected the stability of the rhizosphere microbial community network

A co-occurrence network is a representation of microbial flora and their interactions. Interrelationships among these organisms play an essential role in maintaining microbial communities' structure and functionality across various environments (Zhou et al., 2021). The presence of a significant number of links between microbial taxa enhances network cohesion, while increased connectivity and network interactions play a crucial role in promoting stability within the microbial communities (Morrien et al., 2017). We found that the relationships between taxa in bacterial communities were closer and more complex than those in fungi communities. However, there were very slight differences between bulk soil and rhizosphere soil. In detail, the network complexity and size showed larger changes in healthy rhizosphere soil compared to diseased rhizosphere soil. Therefore, PVY infection weakened the selection of plant roots for microbial communities and increased their network complexity and size. Therefore, PVY infection resulted in a higher degree of connectivity among microbial taxa in the rhizosphere, enhancing rhizosphere microbial community stability. The network degree of taxa within the fungal community exhibited lower values compared to those within bacterial communities, possibly attributable to dissimilarities in the anticipated metabolic activities of these organisms. In response to alterations in environmental conditions and the abundance of other taxa, archaea and bacteria generally demonstrate more prompt reactions than eukaryotes, including fungi (Paul, 2007). Fungi possess the ability to enzymatically degrade intricate organic matter extracellularly, thereby diminishing their obligatory reliance on bacterial and archaeal taxa (Pivato et al., 2008). This characteristic may elucidate the relatively reduced number of correlative interactions observed among fungi.

Microbial taxa were shown to have cooperative and competitive relationships in the interaction network via positive and negative edges (Price et al., 2021). Our results showed that fungal networks were dominated by positive links, whereas bacterial networks showed more negative links. Similar to other studies, the fungal community may adapt to soil physicochemical properties through cooperative symbiosis or mutualism (Fan et al., 2018; Peng et al., 2023). Topologically, network hubs and connectors can be seen as regulators, mediators, or adaptors, while module hubs can be considered integral components within specific modules (Han et al., 2004). In this study, we have classified these hubs and connectors as key species, acknowledging their crucial roles in network structure and their potential as targets for microbial modulation aimed at enhancing crop productivity (Olesen et al., 2007). Bacterial biomarkers, *Roseiflexaceae* and *Sphingomonas*, were found to be connectors in bacterial networks. Fungal biomarkers *Ascomycota_unidentified_1_1* and *Rhodotorula* were also identified as connectors in fungal networks. In addition, subnetworks of biomarkers further indicated their changes among bulk, healthy rhizosphere, and diseased rhizosphere soils through greater competitive ability and more complex interaction. The proportion of negative links and the link numbers of *Roseiflexaceae, Sphingomonas*, and *Sphingobium* with other taxa were the highest in the BCK, BHS, and BIS networks, respectively. In the fungal network FIS, the links between biomarkers and other taxa were dominated by negative links.

### The bacterial communities of bulk and rhizosphere soils were more sensitive to soil physicochemical properties than fungal communities

The composition and structure of rhizosphere microorganisms are influenced by intricate soil properties (Reinhold-Hurek et al., 2015). Mantel analysis revealed that the factors influencing bulk and rhizosphere soils differed between bacterial and fungal communities. Compared to fungal communities, we found more key underlying factors in bacterial communities. In detail, WC, AP, TP, and TK were all key factors affecting bacterial community structure and diversity. However, fungal community diversity was affected by pH and TP, and there was no factor found to significantly affect community structure. This discovery aligns with previous research findings (Ai et al., 2018; Xue et al., 2022). This may be because bacterial and fungal communities have different influence mechanisms for their different characteristics, such as strategies and ecological niches. The impact of WC on the structure and diversity of bacterial communities was evident; however, it had no significant effect on fungal communities. A significant influence of pH was observed in the diversity of the fungal community but not in the bacterial community. A comparable outcome was also documented, indicating that alterations in soil water content had a notable impact on the diversity of rhizosphere bacterial communities, while no significant impact was observed on the composition of the soil, rhizosphere, and root endosphere fungal communities (Naylor et al., 2017). Additionally, the contents of TP, TK, AP, and AK were identified as crucial factors influencing bacterial community diversity, while AP was found to influence fungal community diversity. Mantel analysis also indicated that the key factors affecting the biomarkers of microbial communities were different between bulk, healthy rhizosphere, and diseased rhizosphere soils. WC and pH were key factors affecting the biomarkers of bacterial rhizosphere communities, whereas the biomarkers of bulk bacteria were significantly affected by soil nutrients (e.g., TP and TK). This observation can be attributed to the rhizosphere environment's elevated nutrient conditions compared to the bulk soil, which offers more favorable resources for microbial activities and plant growth (Peng et al., 2022). In addition, the rhizosphere soil of the diseased plant provided higher restricting factors for the bacterial community, thus the biomarkers of *Sphingobium* in BIS showed closer relationships with soil properties. Therefore, our research indicates that soil physicochemical properties exerted a greater influence on bacterial communities compared to fungal communities, and the factors influencing bulk soil and rhizosphere soil differed.

## Conclusion

Our findings demonstrate that soil environment properties significantly impacted soil microbiomes, particularly the bacterial community, while PVY infection did not significantly affect the structure and diversity of rhizosphere bacterial and fungal communities. LEfSe analysis further indicated the significant differences in community compositions and identified the biomarkers among bulk, healthy rhizosphere, and diseased rhizosphere soils. Bacterial networks were more complex and compact than fungal networks. PVY infection increased rhizosphere microbial community stability and resistance to environmental stress through greater connectedness among microbial taxa. Mantel analyses further revealed that WC played an apparent influence on bacterial community structure and diversity, and pH showed significant effects on fungal community diversity. WC and pH were key factors affecting the biomarkers of bacterial rhizosphere communities, whereas the biomarkers of bulk bacteria were significantly affected by soil nutrients, especially for the biomarkers of *Sphingobium*. In summary, this study explored the bacterial and fungal adaptation strategies from bulk soil to rhizosphere soil in healthy and diseased plants, and regulation of community composition and interaction networks were the main adaptation strategies to Hs and IS.

## Data availability statement

The datasets presented in this study can be found in online repositories. The names of the repository/repositories and accession number(s) can be found in the article/Supplementary material.

## Author contributions

YD: Conceptualization, Writing—original draft. WK: Writing—review & editing. XZ: Writing—review & editing. YZ: Writing—review & editing. TX: Writing—review & editing. MC: Writing—review & editing. LZ: Writing—review & editing. JS: Writing—review & editing. ZZ: Writing—review & editing. CC: Writing—review & editing. CZ: Writing—review & editing. HY: Writing—review & editing. SH: Writing—original draft. YG: Writing—original draft.
