# Supplementary material for: Rhizosphere microbial community enrichment processes in healthy and diseased plants: implications of soil properties on biomarkers
Source: Front Microbiol. 2024 Feb 29;15:1333076. doi: 10.3389/fmicb.2024.1333076 (PMC10949921; doi:10.3389/fmicb.2024.1333076)
Supplement: Supplementary file 3 [file Table_3.docx]

**Table S1.** Topological properties of bacterial and fungal networks at ASV level in bulk, healthy rhizosphere and diseased rhizosphere soils. BCK, bulk bacterial network; BHS, healthy rhizosphere bacterial network; BIS, diseased rhizosphere bacterial network; FCK, bulk fungal network; FHS, healthy rhizosphere fungal network; HIS, diseased rhizosphere fungal network.

| **Treatments** | **BCK** | **BHS** | **BIS** | **FCK** | **FHS** | **FIS** |
| --- | --- | --- | --- | --- | --- | --- |
| Nodes number | 1030 | 720 | 777 | 322 | 170 | 146 |
| Links number | 8656 | 4497 | 4215 | 2124 | 844 | 361 |
| Average degree | 16.808 | 12.492 | 10.849 | 13.193 | 9.929 | 4.945 |
| Average path length | 4.674 | 5.583 | 2.35 | 3.501 | 1.392 | 1.204 |
| Average clustering coefficient | 0.791 | 0.756 | 0.751 | 0.971 | 0.993 | 0.991 |
| Betweenness centralization | 0.012 | 0.025 | 0.004 | 0.033 | 0.007 | 0.001 |
| Degree centralization | 0.063 | 0.091 | 0.05 | 0.077 | 0.101 | 0.069 |
| Modularity | 0.812 | 0.804 | 0.875 | 0.808 | 0.753 | 0.857 |
